# Flexible Li[Li_0.2_Ni_0.13_Co_0.13_Mn_0.54_]O_2_/Carbon Nanotubes/Nanofibrillated Celluloses Composite Electrode for High-Performance Lithium-Ion Battery

**DOI:** 10.3389/fchem.2019.00555

**Published:** 2019-08-06

**Authors:** Yan Li, Han Zhang, Zhe Xiao, Renheng Wang

**Affiliations:** International Collaborative Laboratory of 2D Materials for Optoelectronics Science and Technology of Ministry of Education, Institute of Microscale Optoelectronics, Shenzhen University, Shenzhen, China

**Keywords:** lithium ion battery, flexible electrode, Li[Li_0.2_Ni_0.13_Co_0.13_Mn_0.54_]O_2_, carbon nanotubes, nanofibrillated celluloses

## Abstract

Rapidly-growing demand for wearable and flexible devices is boosting the development of flexible lithium ion batteries (LIBs). The exploitation of flexible electrodes with high mechanical properties and superior electrochemical performances has been a key challenge for the rapid practical application of flexible LIBs. Herein, a flexible composite electrode was prepared from the mixed solutions of Li[Li_0.2_Ni_0.13_Co_0.13_Mn_0.54_]O_2_ (LLOs), carbon nanotubes(CNTs), and nanofibrillated celluloses (NFCs) via a vacuum filtration method. The resulting LLOs/CNTs/NFCs electrode delivered an initial discharge capacity of 253 mAh g^−1^ at 0.1 C in the voltage range from 2.0 to 4.6 V, and retained a reversible capacity of 178 mAh g^−1^ with 83% capacity retention after 100 cycles at 1 C. The LLOs/CNTs/NFCs electrode exhibited excellent flexibility along with repeated bending in the bending test. The LLOs/CNTs/NFCs electrode after bending test remained a discharge capacity of 149 mAh g^−1^ after 100 cycles at 1 C, and the corresponding capacity retentions was 76%. The excellent electrochemical performance and high flexibility can be ascribed to the framework formed by CNTs with high conductivity and NFCs with good mechanical properties. The results imply that the as-fabricated electrode can be a promising candidate for the flexible LIBs.

## Introduction

Along with the rise and development of the wearable and flexible electronic products, the concept of flexible devices emerges at the right moment and engulfes the entire world (Manthiram et al., 2008; Kim and Cho, 2010; Agnès et al., 2014). One of the biggest obstacles to the application of flexible electronic products is the development of flexible electrochemical energy storage devices (Ju et al., 2014; Li et al., 2014). LIBs have unique advantages when applied to electronic devices due to its light weight, high specific power, high energy density, and excellent electrochemical performance (Ding et al., 2011; Dunn et al., 2011; Strong et al., 2012; Yan et al., 2013; Yuan et al., 2014; Nitta et al., 2015; Li et al., 2017a,b; Yang et al., 2019). However, the separation of electrode materials and collector fluid affects the electrochemical properties when fold the traditional rigid LIBs, and even lead to short circuit and other serious safety problems (Hu and Sun, 2014; Zhou et al., 2014; He et al., 2016). Therefore, it is of great significance for the development of flexible electronics to endow LIBs with flexibility and develop flexible electrode with excellent electrochemical performance. A well-known strategy to prepare flexible electrode is adopting flexible organic substrates such as polymer and textile instead of traditional copper/aluminum foil as current collector for carrying the active materials (Wang et al., 2006, 2008; Chen et al., 2007; Gao et al., 2015; Huang et al., 2016; Zhen et al., 2016). However, these non-electroactive substrates with low conductivity will greatly affect the quick charge-discharge process of batteries. Therefore, flexible electrodes with conductive matrix such as graphite paper and carbon nanotube film have become a hot research topic, while the weak binding force between active materials and current collector will inevitably result in serious capacity fading and limit further applications (Chou et al., 2008; Xin et al., 2011; Hu et al., 2013). Recently, many researchers had proposed to build the 3D thin-film electrodes which compounding the traditional active electrode material with the conductive carbon-based material and flexible matrix. The carbon-based materials acted as the basic element of the conductive network, and the flexible matrix served on the supporting framework of the whole electrode. This method can fully combine the advantages of carbon-based materials and traditional active electrode materials (Su et al., 2010; Tai et al., 2012; Lian et al., 2014; Yu et al., 2014; Wang et al., 2017b).

Lithium-rich layered oxides (LLOs) have been extensively investigated owing to their high reversible capacity, high working voltage, high energy density, lost cost, and amity to environment (Wang et al., 2009; He et al., 2013; Hu et al., 2018). With a reversible capacity of 250 mAh g^−1^, LLOs were attractive enough to high-energy LIBs and have demonstrated enormous potential in terms of electric vehicles and smart grid applications. As one of the most abundant renewable polymers, nanofibrillated cellulose (NFC) has several advantages such as nanoscale diameter, high homogeneity, high tensile strength and high Young modulus. The one dimension nano-structure is beneficial to bind active electrode particles in the nanometer scale, and allows for the formation of thinner films with higher space efficient (Ho et al., 2011). NFC with excellent mechanical properties can be utilized as flexible matrix and binder for composite electrodes, and has great prospects in flexible energy storage devices. For example, a flexible NFC/LiFePO_4_/Super-P paper electrode was prepared by filtration process. NFCs acted both as electrode binder material and the separator material. The resulting thin electrode was very strong which can withstand of strength up to 5.6 Mpa. The flexible electrode delivered a reversible capacity of 146 mAh g^−1^ and an energy density of 188 mWh g^−1^ of full paper battery at 0.1 C (Leijonmarck et al., 2013). Recently, Wang et al. developed a flexible LiFePO_4_/graphene/NFC electrode with the mechanical support of NFC, and the composite electrode shows high flexibility as well as excellent electrochemical properties (Wang et al., 2018). Carbon-based materials with high conductivity were highly desirable for high-performance LIBs. Many studies have proved that CNTs can improve the electronic conductivity of electrode and offer the buffer spaces for the volume expansion of active material during the cycling process (Chen et al., 2007; Fu et al., 2013; Kang et al., 2016). Thanks to the graphitic structure and good electrical conductivity, CNTs has the capability of forming conductive network structure and is very suitable for preparing self-supporting film electrodes (Bai et al., 2015; Wan et al., 2015). For instance, a binder-free LiMn_2_O_4_/CNT electrode with good flexibility were fabricated by *in-situ* hydrothermal growth for flexible LIBs, and the flexible electrode showed good cycling and rate performance (Jia et al., 2011).

Herein, a flexible LLOs/CNTs/NFCs composite electrode was fabricated via a facile vacuum filtration route. The resulting LLOs/CNTs/NFCs composite electrode exhibited good electrochemical performance as well as high flexibility, which can be ascribed to the three-dimensional conductive framework formed by NFCs and CNTs.

## Experimental

### Synthesis and Characterization

**Figure 1:**
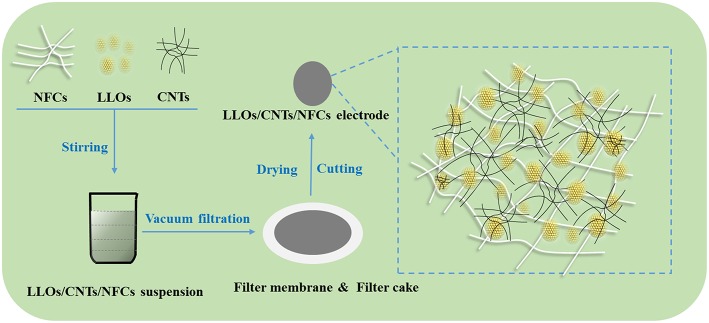
Flow diagram of the preparation procedure of flexible LLOs/CNTs/NFCs electrode.

LLOs were prepared from the Mn_0.54_Ni_0.13_Co_0.13_(CO_3_)_0.8_ precursors (Ningbo Institute of Industrial Technology, CAS.) and lithium carbonate via solid state reaction. Commercially available CNTs (wt % > 95%, Shanghai Macklin Biochemical Co., Ltd.) and NFCs (solid content = 5%, width = 5–20 nm, length ≈ 400 μm, Ningbo Rouchuang Nanotechnology Co., Ltd.) were used without further processing. As shown in Figure 1, the flexible LLOs/CNTs/NFCs electrode was fabricated via a facile vacuum filtration route. Proper amount of LLOs, CNTs and NFCs with a mass ratio of 8:1:1 were dispersed in 100 ml ethanol and then stirred for 20 h, the resulting LLOs/CNTs/NFCs suspension was filtered using a PTFE membrane (0.22 lm pore size, Tianjin Jinteng Experiment Equipment Co., Ltd.). At last, the LLOs/CNTs/NFCs electrode was prepared from the filter cake after drying and cutting. The diameter of the obtained electrode was 10 mm and the weight of the active materials was about 8 mg.

For comparison, the traditional LLOs electrode with aluminum foil as current collector was also prepared according to the procedures reported earlier (Li et al., 2018).

Morphologies of the samples were investigated with scanning electron microscopy (SEM, Gemini300). The structure of the LLOs/CNTs/NFCs electrode were determined by X-ray diffraction (XRD, Bruker D8) equipped with Cu Kα radiation ranging from 10° to 80° at a scan rate of 10°·min^−1^. Raman spectroscopy (FSRS, Renishaw in Via Reflex) was carried out to analysis of the electrode using a laser wavelength of 532 nm.

### Electrochemical Measurement

The as-prepared LLOs/CNTs/NFCs electrode and the traditional LLOs electrode acted as the working electrode for the electrochemical measurements of LIBs. Li metal foil, microporous polypropylene film and the liquid organic electrolytes [1 M LiPF_6_ in EC/DMC/EMC (1:1:1 by volume)] were used as the counter electrodes, separator and electrolyte, respectively. The charge/discharge cycling was carried out in the potential range of 2.0–4.6 V using a CR2032 coin-type cell. The LLOs/CNTs/NFCs electrodes were bended repeatedly with 5 mm curvature radius for different times. The electrochemical performances of the LLOs/CNTs/NFCs electrodes after cyclical bending were tested to investigate the application prospects for the flexible electrode. Electrochemical impedance spectroscopy (EIS) was conducted using a CHI660e electrochemical workstation in the frequency range of 10^−2^-10^5^ Hz.

## Results and Discussion

**Figure 2:**
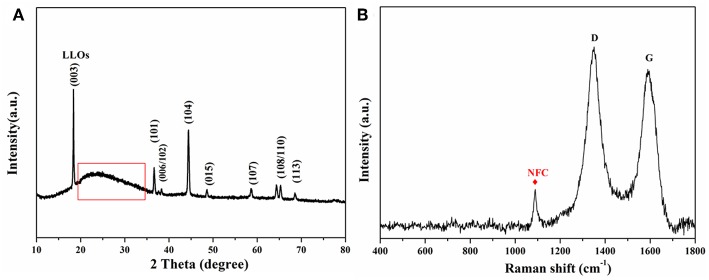
**(A)** XRD patterns and **(B)** FSRS of the LLOs/CNTs/NFCs.

Figure 2A shows the XRD patterns of the flexible LLOs/CNTs/NFCs electrode. The sample was consistent with the well-crystallized LLOs which adopts a hexagonal α-NaFeO_2_ type structure. It's worth noting that the characteristic peak (marked by the red box) can be assigned to the (002) peak of NFCs (22.5°) and CNTs (Sassi and Chanzy, 1995; Tessonnier et al., 2009). FSRS in Figure 2B shows two distinct Raman peaks at 1350 and 1588 cm^−1^, which corresponded to the D-band and G-band of CNTs, respectively (Dresselhaus et al., 2007). The other evident peak centered at 1,095 cm^−1^ was assigned to the asymmetric stretching vibration mode of C–O in the NFCs (Leijonmarck et al., 2013). These results revealed that the as-prepared electrode was composed of LLOs and small amount of NFCs and CNTs, and the crystalline structure of LLOs were maintained well in the composite electrode. It can be expected that the introduction of NFCs and CNTs could enhance the overall flexibility and conductivity of the composite electrode.

**Figure 3:**
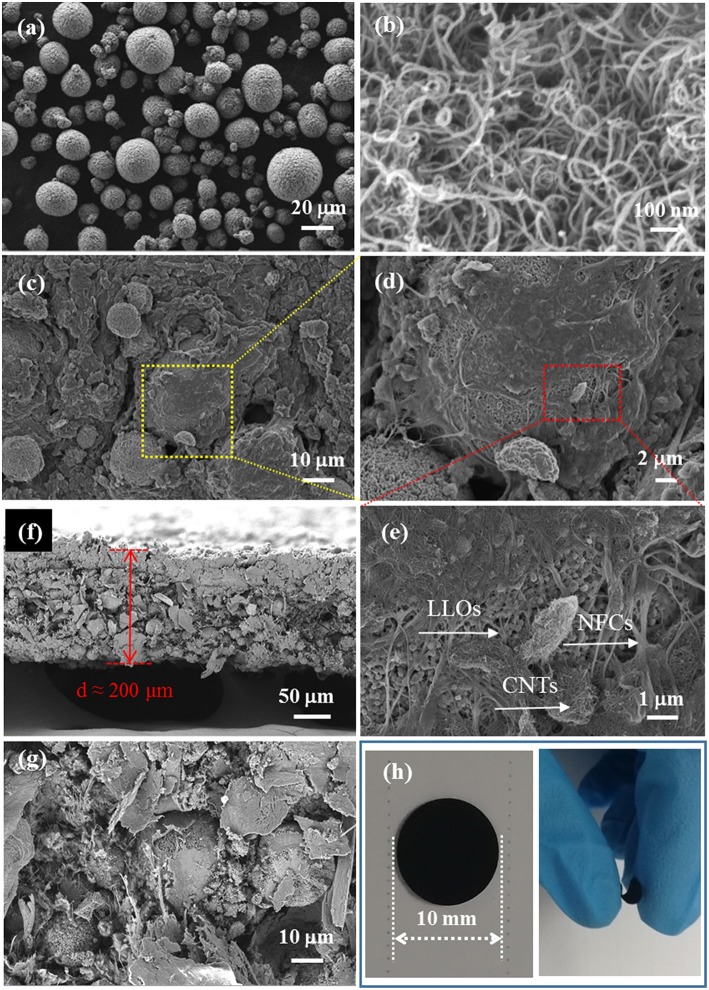
SEM images of **(a)** LLOs, **(b)** CNTs, **(c–e)** LLOs/CNTs/NFCs electrode; **(f,g)** Cross-section images of the LLOs/CNTs/NFCs electrode; **(h)** the photos of LLOs/CNTs/NFCs electrode before and being bending.

SEM images of the samples and photographs of the LLOs/CNTs/NFCs electrode were presented in Figure 3. Figure 3a shows the morphology of LLOs particles, the particles have a micro-grade spherical shape with a diameter ranging from 5 to 20 μm, and the microspheres were formed by numerous primary particles. SEM image in Figure 3c indicates that CNTs remained a regular nanotube shape with a diameter in the range of 10–20 nm. Figures 3c,d shows the SEM images of the LLOs/CNTs/NFCs electrode in different magnification. The LLOs particles, CNTs and NFCs could be clearly observed in Figure 3e. It was evident that the micro-sized LLOs particles were tightly compacted and agglutinated by the frame of NFCs and CNTs, and thus forming the flexible composite electrode. The cross-section images in Figures 3f,g indicate that the thickness of the flexible electrode was about 200 μm, and the LLOs particles, CNTs and NFCs were uniform mixed and distributed in the flexible electrode. The photos of the LLOs/CNTs/NFCs electrode with a diameter of 10 mm illustrate its structural integrity and high flexibility, as seen in Figure 3h. The surface of the LLOs/CNTs/NFCs electrode was verified to be smooth and level. The structural integrity the electrode can be attributed to the entanglement of NFCs and CNTs. The photo of the electrode being bending illustrates that the as-prepared LLOs/CNTs/NFCs electrode demonstrated good flexibility.

**Figure 4:**
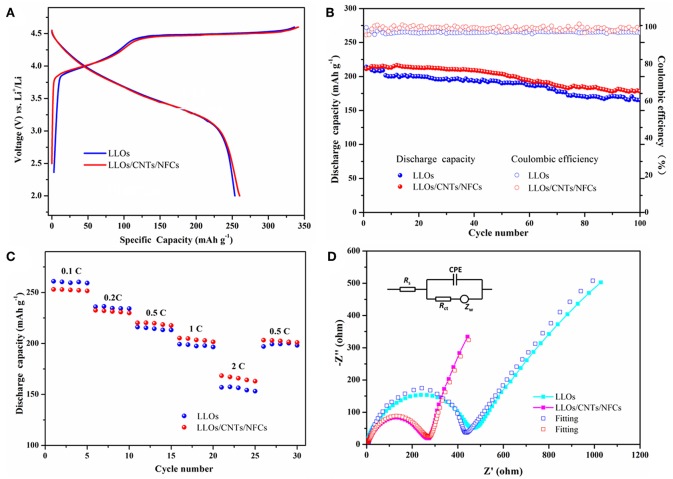
Electrochemical performances of the traditional LLOs electrode and LLOs/CNTs/NFCs electrode: **(A)** initial charge–discharge profiles at 0.1C rate; **(B)** Cycle performances at 1 C rate and the corresponding Coulombic efficiency; **(C)** Rate performances in the rate range of 0.1–2 C; **(D)** EIS plots of two samples.

The electrochemical performances of the traditional LLOs electrode and LLOs/CNTs/NFCs electrode were presented in Figure 4. Figure 4A shows the first cycle curves of the two electrodes at 0.1C rate, the traditional LLOs electrode and the LLOs/CNTs/NFCs electrode exhibite discharge capacities of 253 and 259 mAh g^−1^, respectively. They show a similar initial coulombic efficiency of about 76%. The cycle performances of the two electrodes were presented in Figure 4B. After 100 cycles at 1 C rate, the discharge capacities of the traditional LLOs electrode and the LLOs/CNTs/NFCs electrode remain 165 and 178 mAh g^−1^, respectively. The corresponding capacity retentions were 77 and 83%, respectively. Also notice that both the two samples exhibite nearly 100% coulombic efficiency. As depicted in Figure 4C, the LLOs/CNTs/NFCs electrode shows better rate capability than that of the traditional LLOs electrode. When the current density was 2 C, the discharge capacity of the traditional LLOs electrode and the LLOs/CNTs/NFCs electrode were 157 and 168 mAh g^−1^, respectively. The results indicate that the flexible LLOs/CNTs/NFCs electrode exhibites better reversibility and superior rate capability than the traditional LLOs electrode under the same condition. Figure 4D shows the EIS plots, the fitting results and the corresponding equivalent circuit of the two samples. The curves consist of a semicircle and a straight line. The semicircle in the high and medium frequency can be explained as the charge transfer resistance (R_ct_) and double layer capacitance (C_dl_), and the straight line in the low frequency was a representation of the Warburg impedance Z_w_ (Wu et al., 2019). By comparing the two curves, it was found that the traditional LLOs electrode has higher interfacial impedance compared with the LLOs/CNTs/NFCs electrode. The lower impedance of the flexible LLOs/CNTs/NFCs electrode can be attributed to fast charge transfer and high electrochemical activity provided by the continuous conductive network. This also demonstrates the reasons for the better cycle and rate performances of the LLOs/CNTs/NFCs electrode.

**Figure 5:**
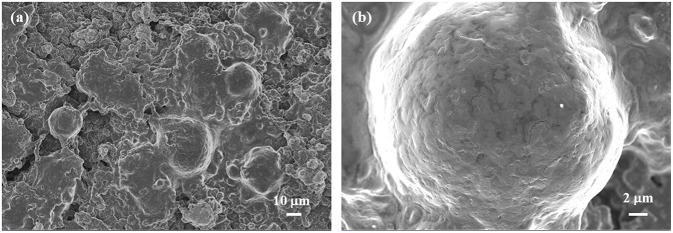
**(a)** Low magnification and **(b)** high magnification SEM images of the LLOs/CNTs/NFCs electrode after 100 cycles at 1C.

The SEM images of the LLOs/CNTs/NFCs electrode after 100 cycles at 1 C were illustrated in Figure 5. The low-magnification image in Figure 5a reveals that the integrity of the electrode was maintained well and the LLOs particles keeped the original spherical shape. It was clear from Figure 5b that the dense and uniform SEI film was covered to the surface of LLOs microspheres (Wang et al., 2017a). The result indicates the dominant structure stability of the LLOs/CNTs/NFCs electrode, and further proves the foregoing reasons for the good electrochemical performances of the flexible LLOs/CNTs/NFCs electrode.

**Figure 6:**
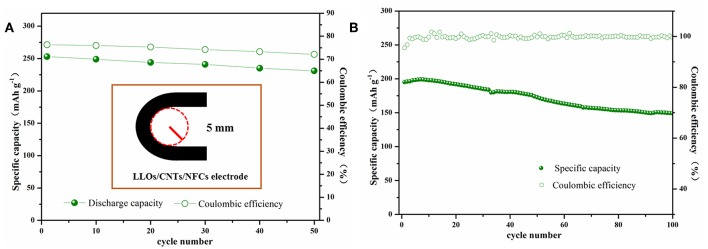
**(A)** The initial discharge capacities and Coulombic efficiencies of flexible LFP/G/NFC electrode vs. bending times, and the inset represents the schematic of the bending test; **(B)** Cycle performance and Coulombic efficiencies of the LLOs/CNTs/NFCs electrode after bended repeatedly below a curvature radius of 5 mm for 50 times.

In order to investigate the practical prospect of the flexible LLOs/CNTs/NFCs electrode, electrochemical performances of the flexible electrode were measured after bending. As exhibited in Figure 6A, the LLOs/CNTs/NFCs electrode was bent with a 5 mm curvature radius for 0–50 times. For the LLOs/CNTs/NFCs electrode, the discharge capacity and coulombic efficiency in the first cycle at 0.1 C decreased with the increase of bending times. The incipient discharge capacity of the flexible electrode after 50 times bending was 231 mAh g^−1^, and the capacity retention rate was 91% after bending for 50 times. At the same time, the initial coulombic efficiency reduces from 76 to 72% after 50 times bending. The cycle performance and coulombic efficiency of the LLOs/CNTs/NFCs electrode after bending for 50 times was depicted in Figure 6B. After 100 cycles at 1 C, the LLOs/CNTs/NFCs electrode delivered a discharge capacity of 149 mAh g^−1^, the corresponding capacity retentions was 76%, and the coulombic efficiency keeped nearly 100%. These tests manifest that the as-prepared LLOs/CNTs/NFCs electrode had high structure stability and flexibility, and the electrochemical properties declined slight after repeated bending. In the LLOs/CNTs/NFCs electrode, NFCs with extraordinary mechanical properties is beneficial to bind active electrode particles and form a flexible freestanding electrode (Nakagaito and Yano, 2004; Aulin et al., 2012). CNTs with superior mechanical properties and electrical conductivity can greatly enhance the mechanical strength and electrochemical activity of the active materials (Hu et al., 2012; Peng et al., 2014). Therefore, the improvement of mechanical and electrochemical performances of the LLOs/CNTs/NFCs electrode can be attributed to the continuous conductive network constructed by NFCs and CNT.

## Conclusions

In summary, the flexible LLOs/CNTs/NFCs electrode was constructed via the vacuum filtration method. The resulting LLOs/CNTs/NFCs electrode exhibited enhanced initial discharge capacity, superior cycle stability and improved rate performance in contrast to the traditional LLOs electrode. The LLOs/CNTs/NFCs electrode exerted high flexibility during the repeated bending processes, and the electrochemical performance performance degraded less after bending. The good electrochemical performances and flexibility can be attributed to the conductive framework formed by NFCs with good mechanical properties and CNTs with high conductivity. This study provides a potential strategy to fabricated flexible electrode for LIBs with good mechanical properties and electrochemical performances.

## Data Availability

All datasets generated for this study are included in the manuscript/supplementary files.

## Author Contributions

YL and RW designed and engineered the samples. YL performed the experiments. YL, HZ, ZX, and RW performed the data analysis. YL and RW wrote the paper. All authors contributed to the theoretical analysis and the general discussion.

### Conflict of Interest Statement

The authors declare that the research was conducted in the absence of any commercial or financial relationships that could be construed as a potential conflict of interest.
